# EXPANDING REACH AND SCALABILITY OF EVIDENCE-BASED FALL PREVENTION PROGRAMS (ERASE FALLS)

**DOI:** 10.1093/geroni/igae098.1583

**Published:** 2024-12-31

**Authors:** Sara Pappa

**Affiliations:** Marymount University, Arlington, Virginia, United States

## Abstract

In 2016, Marymount University was awarded the first Evidence-Based Falls Prevention Programs (EBFPP) cooperative grant by the Administration for Community Living, initiating the Northern Virginia Falls Prevention Alliance. Subsequent funding supported the Academic-Community Partnership for Falls Prevention, addressing the critical need in Northern Virginia—a diverse region with over 250,000 older adults. From 2016 to 2021, our programs reached 5,060 individuals across 75+ sites, through initiatives like Stay Active and Independent for Life (SAIL), A Matter of Balance (AMOB), and the Otago Exercise Program (OEP). We established the innovative MU Regional Training Office (MU-RTO) for affordable EBFPP leader training and support, and have trained over 500 lay leaders in these EBFPP. We adapted to remote delivery during the COVID-19 pandemic, and founded the Northern Virginia Falls Prevention Alliance (NVFPA), growing it to over 135 members. As a founding member of the Virginia Arthritis and Falls Prevention Coalition (VAFPC), we’ve contributed majorly to regional falls prevention. Our efforts include significant research developments, such as the large data evaluation of EBFPPs, housed at the Marymount Center for Optimal Aging (MCOA). The MCOA focuses on aging research with an emphasis on minority inclusion and community-participatory approaches.

